# Drying-Induced Salt Deposition Patterns as a Tool for Label-Free Protein Quantification

**DOI:** 10.3390/bios15080520

**Published:** 2025-08-09

**Authors:** Arturo Patrone-Garcia, Miquel Avella-Oliver, Ángel Maquieira

**Affiliations:** 1 Instituto Interuniversitario de Investigación de Reconocimiento Molecular y Desarrollo Tecnológico (IDM), Universitat Politècnica de València, Universitat de València, 46022 Valencia, Spain; arpatgar@upv.es; 2 Departamento de Química, Universitat Politècnica de València, 46022 Valencia, Spain

**Keywords:** crystallization, precipitation, label-free, imaging, light scattering, evaporation, desiccation, NaCl, naked-eye, droplet

## Abstract

This work reports a label-free analytical strategy based on protein-induced modulation of salt crystallization patterns upon drying. This method relies on the consistent observation that protein-containing saline samples produce distinct salt deposition morphologies compared to protein-free controls. The work first demonstrates the concept of this phenomenon and characterizes the structural features of the resulting salt patterns. Then, systematic experiments with different solution compositions, substrates, surface coatings, and protein types confirm the generality of this differential deposition behavior and its dependence on total protein concentration. Two complementary measurement approaches are evaluated: a custom laser-scattering setup for optical attenuation measurements and a digital image analysis method based on pixel intensity distributions. Both strategies enable quantitative protein detection in simple (casein) and complex (human serum) samples, offering good correlations between signal and concentration and detection limits in the range of 2–18 µg·mL^−1^ for digital image analysis and 162–205 µg·mL^−1^ for optical attenuation measurements. These findings introduce an appealing paradigm for protein quantification exploiting drying-mediated crystallization phenomena, with potential for simple and label-free bioanalytical assays.

## 1. Introduction

Label-free bioanalytical strategies based on solid-phase assays are widely used in biosensing platforms, offering simplicity, cost-effectiveness, and compatibility with point-of-care diagnostics [1,2,3,4]. These assays often involve the interaction of biomolecules with a solid surface, followed by signal generation through optical, electrochemical, or mass-based readouts. Along these lines, physical processes associated with drying and precipitation from saline solutions have drawn increasing attention for their potential to contribute to new signal-generation mechanisms in biosensing [5,6].

In this context, the *salting-out* phenomenon represents a classical example involving proteins and saline aqueous solutions [7,8]. By increasing the salt concentration, protein–solvent interactions are weakened, leading to protein aggregation and precipitation. This technique has long been used in protein purification and is governed by thermodynamic interactions in bulk solution, leading to uniform protein aggregation not associated with surface-localized effects or pattern formation upon drying [9,10].

Additionally, several studies have explored how drying droplets of biological fluids on solid surfaces can generate spatially heterogeneous deposition patterns [5,11,12,13]. These patterns are shaped by a combination of solvent evaporation, capillary flow, surface tension gradients, and interactions between solutes and substrates. When salts are present in the medium, their crystallization during drying can be visibly altered by the presence of proteins in terms of changes in morphology, texture, and spatial distribution of the final deposit [14,15]. These phenomena have been extensively studied in the context of the coffee-ring effect and drying-induced pattern formation [16,17]. Proteins have been shown to influence the morphology of salt deposits during evaporation, leading to differences depending on concentration, sample composition, and substrate properties [14,15].

Several studies have proposed that such deposition patterns may contain diagnostically relevant information. For example, morphological differences in dried droplets of biological samples have been explored for disease classification using imaging and machine learning techniques [18,19,20,21,22]. While such findings indicate an interplay between proteins and crystallizing salts during drying, most reports have focused on qualitative observations or exploratory pattern recognition.

Despite this growing interest, the connection between protein content and salt crystallization during drying remains underexplored from a quantitative point of view. In this paper, we explore drying-induced crystallization phenomena in protein-containing saline solutions and examine how these effects may be leveraged in a biosensing context. In particular, this study focuses on harnessing this phenomenon for straightforward and label-free quantification proteins in liquid samples. Our observations point to consistent and reproducible differences in deposition patterns depending on sample composition, suggesting a potential analytical value beyond visual inspection. Building on this, we investigate the use of optical and image-based strategies to probe these effects with higher sensitivity and quantification potential.

## 2. Materials and Methods

### 2.1. Materials

Phosphate-buffered solution (PBS, 8 mM, Na_2_HPO_4_, 2 mM, KH_2_PO_4_, 137 mM, NaCl, 2.7 mM, KCl, pH 7.4), PBS-T (PBS with Tween 20 0.05% (*v*/*v*)), and carbonate-bicarbonate buffer (15 mM Na_2_CO_3_, 34 mM NaHCO_3_, pH 9.6) were prepared by dissolving them in ultrapure water (Milli-Q, Millipore Iberica, Darmstadt, Germany) and, afterwards, filtering them through 0.45 nylon filters provided by Scharlab (Sentmenat, Spain). Bovine serum albumin (BSA), whole antiserum with anti-BSA specific antibodies produced in rabbit (4.1 mg·mL^−1^ of specific IgGs), goat anti-human antibodies (5.0 mg·mL^−1^ of specific IgGs), casein, ovoalbumin from chicken egg, hemoglobin from bovine blood, β-lactoglobulin from bovine milk, and albumin from human serum were purchased from Sigma-Aldrich (Madrid, Spain). Normal human serum was provided by Merck (Darmstadt, Germany) and ethanol by VWR Chemicals (Radnor, PA, USA). Specific anti-BSA antibodies were obtained by purifying the antiserum with a BSA-activated HiTrap^®^ NHS-activated High-Performance column from Merck (Darmstadt, Germany), where the eluted solutions were then filtered employing Amicon Ultra—0.5 mL 10 K centrifugal filters from Fisher Scientific (Hampton, VA, USA). The glass slides (25 × 75 × 1 mm, premium line) were purchased in Labbox (Barcelona, Spain), the polycarbonate chips (25 × 75 × 1 mm) in Plexi (Valencia, Spain), and the stickers employed for the incubation chambers were cut from a black matte self-adhesive film with a 100 µm thickness provided by Aironfix (Barcelona, Spain).

### 2.2. Assays

In order to prepare the slides for the assay, they were cleaned in an ultrasonic bath immersed in an ethanol 30% solution, for 5 min three times. Afterwards, they were dried by employing a stream of air, and a previously cut incubation chamber made of self-adhesive film (6 assay areas per slide) was adhered to its surface (Figure S1). To coat the slides with BSA, the protein was dissolved in carbonated buffer (50 mg·L^−1^), and the solution was left on the surface overnight at 4 °C. Then, they were rinsed with PBS-T and miliQ water to finally dry them under a stream of air.

To incubate the liquid samples on the slides, two different approaches were performed. On the one hand, liquid samples (2.5, 4 or 5 µL) were dispensed on the incubation chamber and left to interact for 15 min. Afterwards, the slides were tilted in order to remove most of the solution on the slide, dropping it onto paper, and the remaining thin liquid layer on the assay surface after the dropping was let dry at room temperature or at 37 °C. On the other hand, 5 µL of the same solutions were incubated on the surface and let dry at 37 °C.

### 2.3. Microscopy

The morphology of the crystallization patterns at the nanoscale was analyzed by atomic force microscopy (AFM) employed in the non-contact mode in air a Bruker Multimode 8 microscope (Bruker, MA, USA) and with RFESPA probes (MPP-21120-10 Burker, MA, USA). The samples did not need any preparation. The data was then processed with the Nanoscope analysis 1.8 software. Also, a high-resolution field-enhanced scanning electronic microscope (HR-FE-SEM) was employed for the microscopy measurements, using a GeminiSEM 500-8203017153 SEM microscope from Zeiss (Oberkochen, Germany).

In order to prepare the samples for SEM, even though they were non-conductive, no coating was needed. Instead, a colloidal silver solution in toluene provided by Electron Microscopy Science (Hatfield, PA, USA) was employed. The solution was distributed around the well, and it was afterwards let dry, creating a conductive bridge for electron dissipation. The images were acquired by a secondary electron detector and backscattered electron detector. Also, an elemental analysis of the surfaces was carried out by employing an energy-dispersive X-ray spectroscopy (EDX) detector incorporated in the microscope.

### 2.4. Optical Setup

A custom optomechanical setup was built and used to quantify the attenuation of a laser beam produced by the scattering events of the salt deposits on the slide surface generated by the assay. It consists of a red diode laser (MLL-III-642 and PSU-III-LED power supply, Changchun New Industries Optoelectronics Technology Co., Changchun, China). The laser irradiated its beam towards the slide containing the assay, fixed on a sample holder. Then, a PM100D handheld digital power meter with a S120VC photodiode from Thorlabs (Newton, NJ, USA) was set to measure the intensity of the transmitted laser beam.

In order to study the effect of the incidence angle, the sample holder was mounted on a high-precision rotating stage composed by a Motorized Precision Rotation Stage and a K-Cube Brushed DC Servo Motor Controller from Thorlabs (Newton, NJ, USA). 

### 2.5. Imaging Analysis

For the image analysis, photographs of the slides after the assays were acquired with an Olympus SZ61 stereomicroscope with an attached LC20 camera from Evident Scientific (Tokyo, Japan). The images were then transformed into a 16-bit grayscale tiff format with the ImageJ software [23], and then, the same software was used to obtain the histograms of the pixel intensity distribution of each image. Finally, the MS Excel software (MS Excel for Microsoft 365) was used to determine the sum of pixels with intensity above a threshold to be used as the analytical signal. When required, pixel intensity cross-section profiles of the images, as well as the analysis of particle sizes and distribution on the surface, were also obtained with the ImageJ software (version 1.53e) [23].

## 3. Results and Discussion

### 3.1. Differential Salt Deposition

Biorecognition assays, such as immunoassays and DNA hybridization assays, are commonly performed in heterogeneous formats where solutions containing target biomolecules are put in contact with solid surfaces. For biosensing applications, these surfaces are previously functionalized with biomacromolecules and then saline aqueous solutions (samples and buffers) are incubated on them. Both in the functionalization and in the assay, these incubations are performed in many consecutive stages, where the last one is a washing step (typically including water rinsing) to remove the excess of samples and salts that remain on the sensing surface after the assay.

An exploration experiment revealed an interesting precipitation phenomenon taking place when the final washing step was omitted from the assay. This experiment was based on a model immunoassay in label-free format, where anti-BSA antiserum in PBS-T was incubated on glass surfaces previously functionalized with BSA, and the sample was left to dry on the surface after the incubation. As expected, the high amount of salts contained in the PBS-T became deposited on the surface after the assay. But interestingly, this deposition followed a differential behavior depending on the concentration of the target biomacromolecules on the sample.

**Figure 1:**
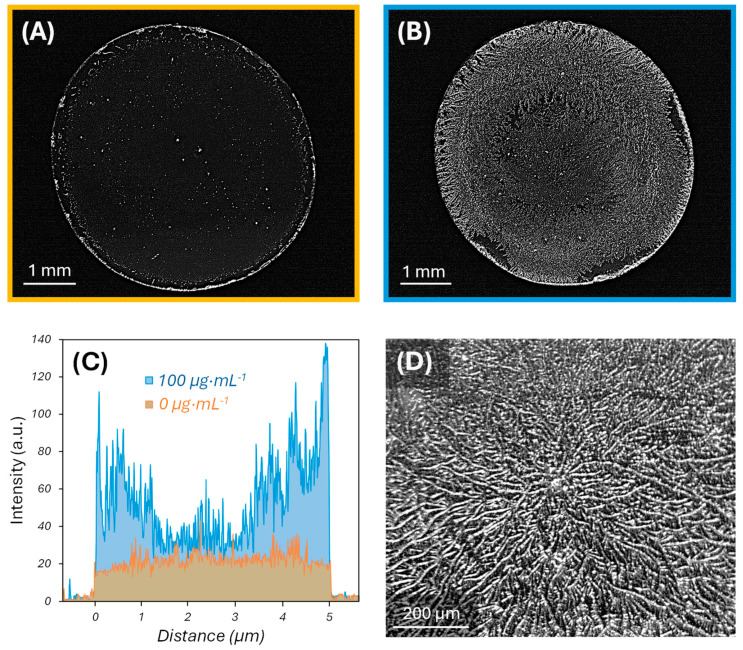
Images under the optical microscope of glass surfaces functionalized with BSA, after the incubation and drying of anti-BSA antiserum with (**A**) 0 and (**B**) 100 µg·mL^−1^ of specific antibodies in PBS-T. (**C**) Pixel intensity cross-section profiles of the optical microscopy images after the incubation and drying of 0 µg·mL^−1^ (orange) and 100 µg·mL^−1^ (blue) of specific antibodies in PBS-T. See Figure S3 for the positions of the cross-sections. (**D**) Zoomed view of a nucleation center under the optical microscope.

This differential deposition can be observed with the naked eye and is clearly revealed under examination with a simple optical microscope. As shown in Figure 1, the morphology and distribution of the deposited salts is significantly different after the incubation and subsequent drying of blank solutions (Figure 1A) compared to anti-BSA antiserum (Figure 1B), both in PBS-T. The first case displays a thin ring of salt deposition confined in an outer perimeter of the assay surface, together with a slight deposition with sparse aggregates in the inner circular area. In contrast, when antiserum in PBS-T is incubated and dried on the surface, the salts become deposited on the whole surface. The optical density is slightly higher also in the outer ring in this second case, but the magnitude of the deposition in the central circle is substantially greater compared to the first case (Figure 1A–C). The surfaces with the BSA coating do not display any saline deposition before the assay (Figure S2), and this trend of differential deposition based on the target concentration was systematically observed in replicated experiments (Figure S3). At the microscale, the deposition of salts after the incubation and drying of solutions containing antiserum follows a dendrimer-like geometry, consisting of nucleation centers from where dendritic structures grow and expand through the surface (Figure 1D).

**Figure 2:**
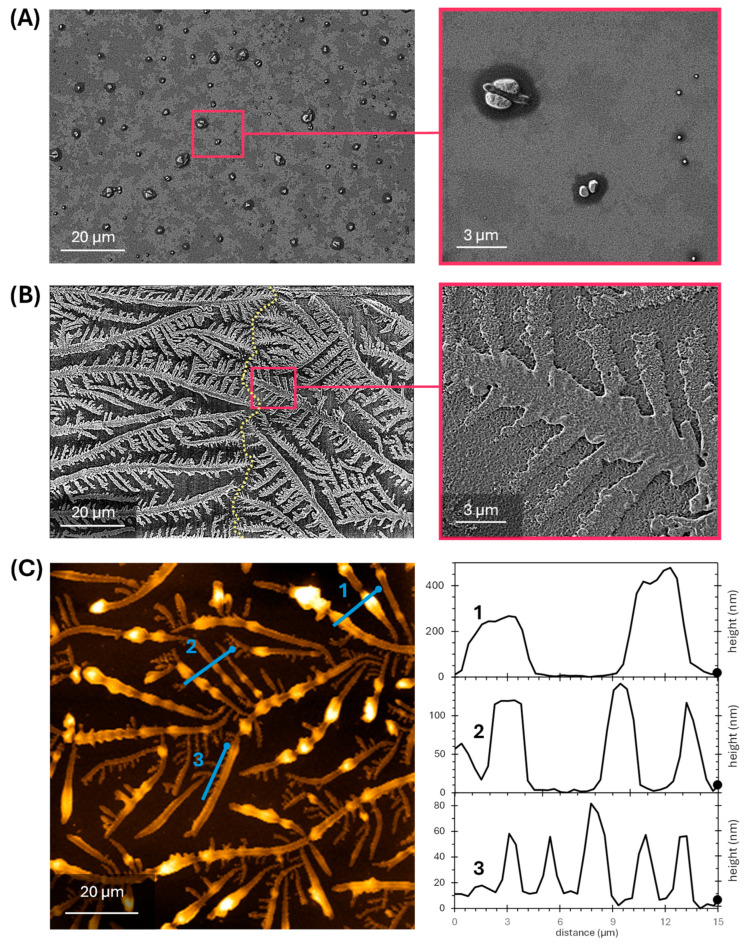
Microscopy characterization. HR-FESEM images of glass surfaces coated with BSA, after the incubation and drying of (**A**) PBS-T and (**B**) anti-BSA antiserum. Both scans in PBS-T and the zoomed view of anti-BSA were captured with a secondary electron detector, whereas the left image of anti-BSA was acquired with a back-scattered electron detector. The vertical dashed line across anti-BSA (left) indicates the dendritic growth boundary. See Figure S4 for larger scans of these FESEM images. (**C**) AFM scans of the dendritic patterns after the incubation of anti-BSA (left) and the corresponding height cross-sections (right graphs). The numbered blue straight lines in the image (left) indicate the position of each height cross-section (shown in the graphs on the right). Note that the thicker dot at the edge of each blue line is also present at the right edge of the corresponding cross-section and indicates its orientation.

The differential deposition behavior depending on the concentration of the target antiserum is also observed when studying the surfaces by scanning electronic microscopy. After the incubation and drying of blank solutions (PBS-T), the electron microscopy scans reveal a distribution of particles spread along the surface (Figure 2A), with around a 28% of them between 0.5 and 3 µm in size and the rest between 50 and 500 nm, and a total surface density about 2900 particles mm^–2^. In contrast, after the incubation and drying of antiserum (100 µg mL^–1^ of anti-BSA in PBS-T), the dendritic pattern on the surface is again revealed at the microscale (Figure 2B). At this level, this pattern is characterized by a main trunk about 2–5 µm wide, with primary branches around 1–2 µm wide, from where secondary branches about 0.5 µm wide also emerge. The self-similarity at different micrometric scales of this geometric structure, also observed at the millimetric scale (Figure 1D), reveals a certain fractal component in the dendritic pattern described by this precipitation phenomenon.

The microscopy images also reveal the dendritic growth boundaries of these patterns, where the growth front of adjacent nucleation centers meets and defines linear interfaces of interrupted dendritic growth, as displayed by Figure 2B [24,25]. Furthermore, this FESEM image was captured using a back-scattered electron detector, where heavier atoms lead to an increased probability of elastic scattering events and appear brighter in the image. This elemental mapping supports the hypothesis that the dendritic patterns are mainly constituted by the inorganic salts of the PBS buffer, since the atomic number of the elements of these salts (K, Cl, P, Na, O, and H) is in average higher than those of the biomolecules (mainly O, N, C, and H) and the glass surface (Si and O).

The same dendritic structure having the trunk and branches geometry and width dimensions discussed above was also observed under AFM examination (Figure 2C). This measurement also reveals that the height of the dendritic structures on the glass surface is about 200–500 nm for the main trunks, 100 nm for the primary branches, and below 100 nm for the secondary branches of the dendritic patterns.

### 3.2. Characterization

After demonstrating the differential deposition behavior depending on the antiserum concentration, and analyzing it at the nanoscale, this section focuses on characterizing this phenomenon by studying the influence that the usual experimental variables of biosensing assays have on this crystallization. The underlying mechanisms of these formation of patterns during salt crystallization are still not fully understood in the literature, and there is an effort in the scientific community to create models to elucidate and predict this phenomenon [5,24,26,27,28,29]. Reports in the scientific literature suggest that the resulting morphology of the deposited salt depends on whether the precipitation process takes place in equilibrium conditions [30,31]. Slowing down the precipitation kinetics brings the conditions closer to the equilibrium and therefore to the formation of lower energy structures like crystals. In contrast, evaporation and faster drying of the solution shift the equilibrium conditions and lead to a diffusion-limited aggregation through which the morphology of the resulting crystal depends on a complex interplay among the involved variables that affect the dynamics of crystal growth [30,31].

Initially, the effect of the sample incubation volume over BSA-coated glass substrates was qualitatively assessed by visual inspection and revealed no significant variation across tested volumes. Notably, assays incubated with 2.5 µL exhibited a slightly reduced signal, and subsequent experiments utilized 5 µL volumes (Figure S5). The drying temperature was then evaluated at 37 °C and room temperature (~20 °C), with a subtle difference observed since drying at 37 °C produced a faint concentric deposition pattern that was particularly evident in blank solution assays (Figure S6). Despite this, both temperatures proved suitable for generating the aimed differential deposition behavior, and 37 °C was selected for the next experiments in this study.

The effect of the sample matrix on the differential deposition phenomenon was then evaluated by varying the medium in which the target antibody was dissolved. As shown in Figure S7, the expected differential deposition behavior was observed in the positive and negative controls solved in PBS-T. However, this phenomenon did not take place when the antibody was dissolved in water. Solutions of NaCl (the main component of PBS-T) at the same concentration present in PBS-T showed precipitation in both the blank and positive assays, indicating the absence of the differential deposition phenomenon. Similar results were obtained with the KCl solutions. Notably, PBS without Tween 20 also failed to produce differential deposition, resembling the behavior seen with NaCl. These findings suggest that both salts and the surfactant contribute synergistically to the differential deposition phenomenon, as it only arises in PBS-T.

Then, the role of surface nature and biolayer composition was investigated. Blank solutions and samples containing anti-BSA were incubated on BSA and HSA biolayers immobilized on glass and polycarbonate substrates. These materials were selected due to their widespread use in heterogeneous bioassays and their different hydrophilicity, allowing assessment of potential polarity effects. In addition to BSA as a probe for anti-BSA IgG and HSA as a negative control, samples were also incubated directly onto uncoated substrates. The results (Figure S8) show no appreciable influence of the substrate (glass or polycarbonate) or biolayer, as all conditions yielded similar outcomes: low precipitation in blank solutions and increased deposition in the presence of antiserum. These findings indicate that the biorecognition between the immobilized probe and the target biomolecule does not drive the observed effect. In fact, the differential deposition was slightly more pronounced on raw surfaces without protein coatings. This suggests that, in addition to the composition of the saline medium discussed above, the key to the phenomenon lies in the nature of the sample, which is explored next.

**Figure 3:**
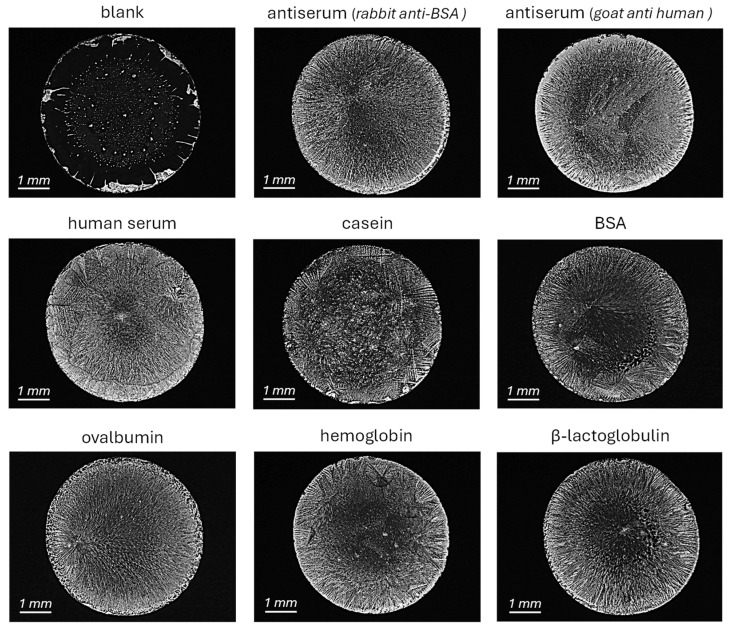
Optical microscopy images for the assays after the incubation and drying of a blank solution (PBS-T) and different protein solutions, all of them at 950 µg·mL^−1^ of total protein in PBS-T over glass surfaces.

To investigate the role of sample composition, assays were performed on raw glass substrates using various protein samples dissolved in PBS-T. As shown in Figure 3, blank solutions produced low precipitation levels as observed before, occasionally with faint deposition localized in the edge of the assay area. The incubation of anti-BSA serum resulted in clear precipitation, while purified IgG at equivalent concentration yielded a similar yet less intense response (Figure S9). Interestingly, incubation of a different antiserum (goat anti-human) at the same total protein concentration produced a comparable signal, as also did conventional human serum and solutions containing individual proteins such as casein, BSA, ovalbumin, hemoglobin, and β-lactoglobulin at the same concentration (Figure 3). Therefore, in all cases, the deposition of salts in the incubation chambers after the assay only took place when the liquid sample contained proteins, regardless of the nature of these proteins. These results indicate that the differential precipitation behavior is not specific to a particular synergy between antiserum and PBS-T, but it potentially is a general response to the presence of proteins. While crystal morphology varied slightly depending on the protein, the differential effect was consistently observed. This suggests that the precipitation magnitude is related to the total protein concentration, offering a simple and label-free approach for protein quantification, which is further explored in the next section.

### 3.3. Quantification

**Figure 4:**
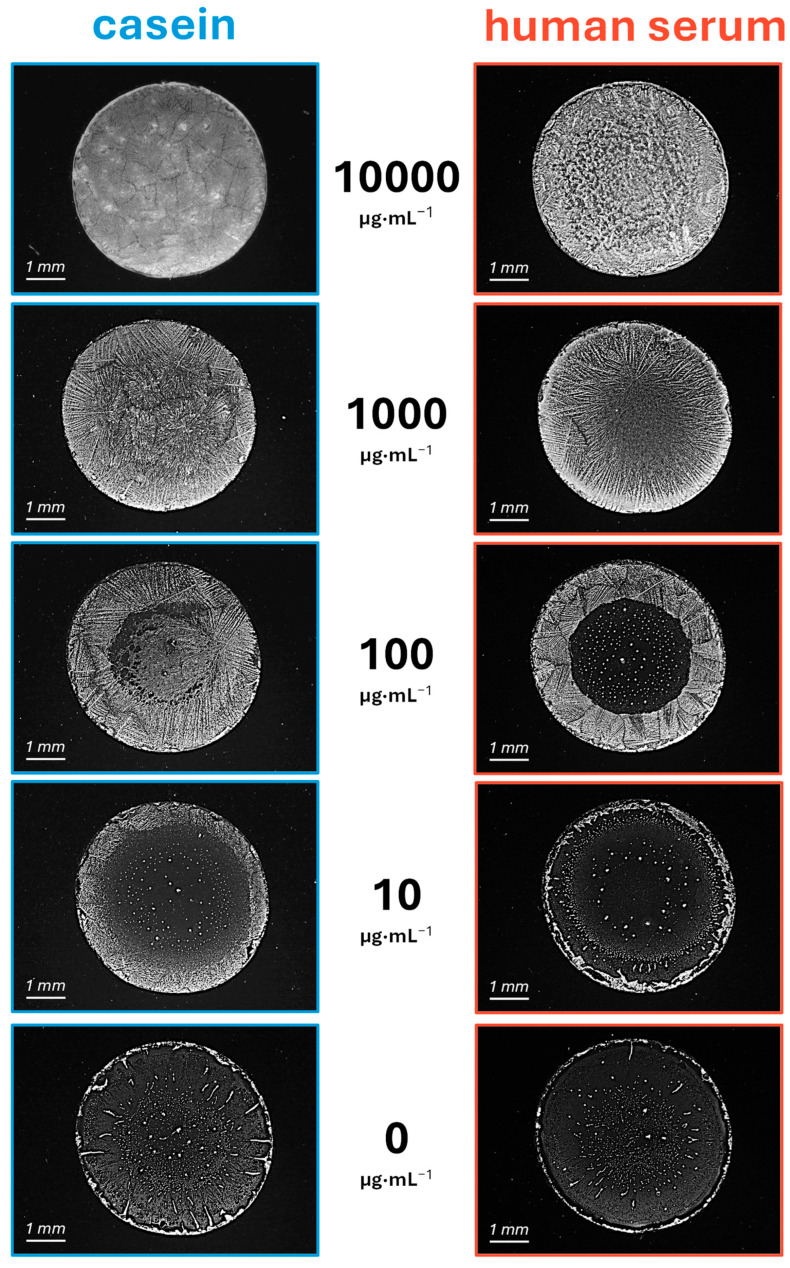
Optical microscopy images of representative assay results after the incubation of casein and human serum at a range of total protein concentrations in PBS-T over glass surfaces.

To evaluate the quantitative response to protein concentration, a range of protein concentrations in PBS-T were tested by incubating and drying samples on raw glass. Casein was used as a model for single-protein samples, while human serum was selected to represent a more complex mixture sample. Visual inspection of the assays revealed that the extent of precipitation increased progressively with protein concentration, as shown in Figure 4. In both cases, this increase followed a radial growth pattern, with salt deposits expanding from the edge toward the center. As concentration increases, a larger portion of the surface becomes covered by these crystalline deposits, as illustrated in Figure S10. This correlation between protein concentration and deposition magnitude was clearly visible in these photographs of the assays and also readily discernible by direct naked-eye visualization. Beyond this qualitative assessment, we next explored the potential of this phenomenon for quantitative analysis using instrumental methods. In particular, we explored two approaches, one based on optical scattering and the other based on image analysis.

**Figure 5:**
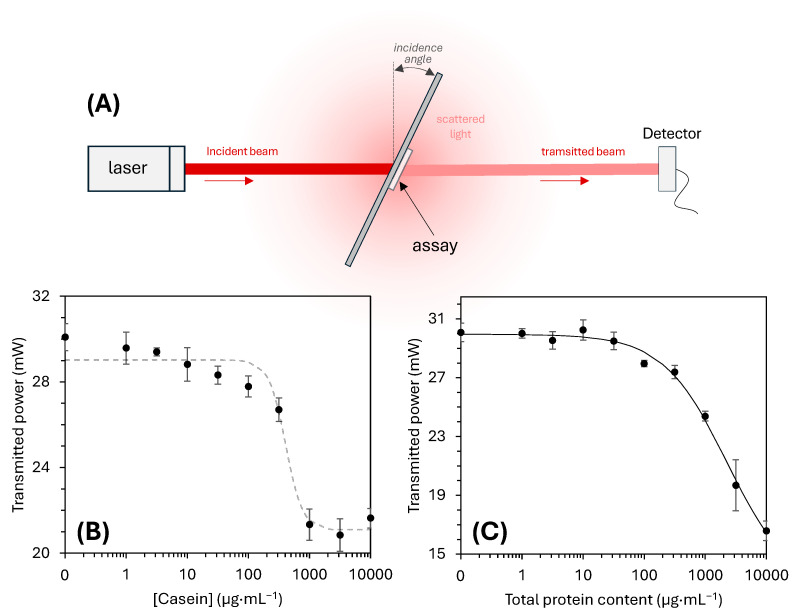
Scattering-based quantification system. (**A**) Scheme of the detection setup. Dose–response curve for (**B**) casein and (**C**) total protein content in human serum.

For the scattering-based method, we developed a custom optomechanical setup designed to detect light dispersion caused by the precipitated deposits on the glass, whose whitish color indicate that they scatter incident light. Since the extent of precipitation increases with the protein concentration, we hypothesized an increase also in the magnitude of scattering events taking place on the slide surface, thus attenuating an incident laser beam and using the intensity of the transmitted light as analytical signal to measure protein concentration. The setup, illustrated in Figure 5A, consists of a laser source illuminating the assay area, and a detector measuring the transmitted light. Greater scattering leads to lower detected intensity and thus stronger attenuation signals. We first optimized the system by evaluating the impact of the incidence angle. Although higher angles increase the optical path length of the laser through the assay zone, the results show no significant trend or enhancement in response (Figure S11A). In contrast, laser power had a marked influence. When varying laser intensity for both the blank and positive assays, the signal increased with the laser power and reached a maximum at 40 mW under the tested configuration (Figure S11B).

This optimized scattering system was then applied to quantify protein concentration in both the casein solutions and human serum over a range of concentrations. As shown in Figure 5B for the casein assay, the transmitted light intensity decreases with the increase in the concentration of this protein in the sample. Although this data presents a clear decreasing trend that agrees with the initial hypothesis based on scattering, its fitting to the sigmoidal curve displays a rather moderate correlation (R^2^ = 0.9687), from which a limit of detection of 205 µg·mL^−1^ and a limit of quantification of 501 µg·mL^−1^ are inferred. The system performed even better with human serum, where a higher correlation (R^2^ = 0.9937) was observed between attenuated optical signal and total protein content (Figure 5C), resulting in a detection limit of 162 µg·mL^−1^ and a quantification limit of 1084 µg·mL^−1^.

**Figure 6:**
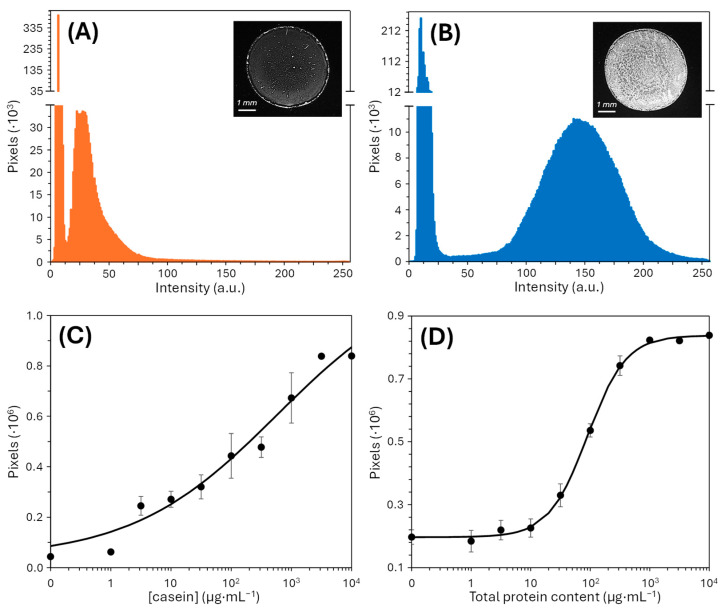
Imaging-based quantification system. Representative histograms for the analysis of (**A**) blank solutions and (**B**) positive samples. Dose–response curve for (**C**) casein and (**D**) total protein content in human serum.

We next focused on quantification based on image analysis. Images of each assay were acquired using an optical microscope, and signal intensity was evaluated by generating histograms representing the distribution of pixel intensities within each image. Histograms for blank solutions were shifted toward lower intensity values (Figure 6A), whereas positive controls exhibited a greater contribution of high-intensity pixels (Figure 6B), as expected. From the observation of these histograms, an intensity threshold of 50 was established, and the analytical signal was defined as the sum of pixels with intensity values equal to or greater than this threshold. The resulting data revealed a correlation between casein concentration and analytical signal, as illustrated by the dose–response curve in Figure 6C. In this initial assay, the correlation was moderate (R^2^ = 0.9679), yielding a limit of detection of 2 µg·mL^−1^ and a limit of quantification of 167 µg·mL^−1^ of casein. In contrast, the analysis of human serum produced a well-defined dose–response curve, with a strong correlation between total protein concentration and analytical signal (R^2^ = 0.9990) when fitted to a classical sigmoidal model (Figure 6D). This response enabled the determination of a detection limit of 18 µg·mL^−1^ and a quantification limit of 58 µg·mL^−1^ of total protein content in human serum.

Contrasting this novel approach to traditional established methodology such as UV/Vis spectrophotometry or mass spectrometry, some advantages and disadvantages can be highlighted. On the one hand, mass spectrometry is a powerful technique with much lower detection limits and unique protein identification capabilities. Nevertheless, this method generally needs sophisticated instrumentation, bulky and expensive, together with highly trained users. Regarding UV/Vis spectrophotometry, although not as powerful as mass spectrometry, it is also a mature technique with higher performance in terms of sensitivity and characterization perspectives when compared to the approach presented in this paper in its current form. Nevertheless, for UV/Vis spectrophotometry, specific, costly equipment is still required, especially for analyzing low volumes of sample. Although the methodology presented in this paper does not reach such lower limits in this first study, its simplicity and low-cost materials represent a significant hallmark, even pointing towards instrumentation-free and naked-eye determination. Beyond the initial demonstration reported in this work, prospective studies to fully characterize the potential of this approach include an in-depth analysis of the role of experimental variables (temperature, volumes, size and height of the incubation chamber, etc.), the effect of potential interferent species and other saline solutions, and the relationship between the nature of the protein and the morphology of the crystallization pattern.

## 4. Conclusions

This study explores the emergence of differential salt precipitation behavior in the drying of protein-containing saline solutions and their analytical potential for protein quantification. The results demonstrate that both the morphology and mainly the magnitude of this salt deposition process are influenced by the total protein content in the sample and nature of the saline solution. This trend was consistently observed across all tested single-protein solutions (bovine serum albumin, casein, ovalbumin, hemoglobin, and β-lactoglobulin) as well as more complex protein mixtures as human serum and antiserums (rabbit and goat), which highlights the general nature of the phenomenon. Moreover, the effect proved robust across different surfaces, including those functionalized with immobilized protein bioreceptors, indicating its compatibility with common biosensing platforms and its independence from surface biorecognition events. Importantly, the extent of salt deposition not only differs clearly between the negative and positive controls but also shows a quantitative correlation with protein concentration. This correlation can be directly appreciated visually with the naked eye, and it can be quantitatively assessed through light scattering measurements using a simple optical setup, or more sensitively through image analysis based on pixel intensity histograms. Beyond this initial demonstration, further research is needed to thoroughly characterize the system, explore its full capabilities, and evaluate its suitability for specific practical applications. Altogether, these findings uncover a reproducible and robust physical effect with promising perspectives for the development of low-complexity, label-free protein quantification strategies based on evaporation-induced crystallization.

## Data Availability

The data presented in this study are available upon request from the corresponding author. The data are not publicly available due to privacy restrictions.

## Supplementary Materials

The following supporting information can be downloaded at: https://www.mdpi.com/article/10.3390/bios15080520/s1, Figure S1: Photograph of the slide’s surface; Figure S2: Optical microscopy images of the six replicates of the glass slide surface before the assay; Figure S3: Optical microscopy images of the replicates in the assay shown in Figure 1; Figure S4: Larger scans of the FESEM images displayed in Figure 2A,B; Figure S5: Optical microscopy images of three replicates of assays after the incubation and drying of different volumes; Figure S6: Optical microscopy images of representative assays after the incubation of antiserum dried at room temperature and in an oven at 37 °C; Figure S7: Optical microscopy photographs of assays after the incubation and drying of antiserum solved in different solutions; Figure S8: Optical microscopy photographs of representative assays after the incubation and drying of antiserum on glass and polycarbonate surfaces coated with BSA, HAS and raw materials without any protein coating; Figure S9. Optical microscopy photographs of representative assays after the incubation of a blank sample, a solution of purified anti-BSA IgG, and anti-BSA antiserum in PBS-T; Figure S10: Optical images of assay results for human serum samples at different total protein concentrations; Figure S11. Optimization results of the scattering-based optical setup.
