# Supplementary material for: Drying-Induced Salt Deposition Patterns as a Tool for Label-Free Protein Quantification
Source: Biosensors (Basel). 2025 Aug 9;15(8):520. doi: 10.3390/bios15080520 (PMC12384144; doi:10.3390/bios15080520)
Supplement: Supplementary file 1 [file biosensors-15-00520-s001.zip › biosensors-3767944-supplementary.pdf]

biosensors

Supplementary Material

Drying-Induced Salt Deposition Patterns as a Tool for Label-free Protein Quantification

Arturo Patrone-Garcia [a], Miquel Avella-Oliver [a,b,*] and Ángel Maquieira [a,b,*]

[a] *Instituto Interuniversitario de Investigación de Reconocimiento Molecular y Desarrollo Tecnológico (IDM), Universitat Politècnica de València, Universitat de València, 46022 Valencia, Spain. e-mail : arpatgar@upv.es*

[b] *Departamento de Química, Universitat Politècnica de València, 46022 Valencia, Spain.*

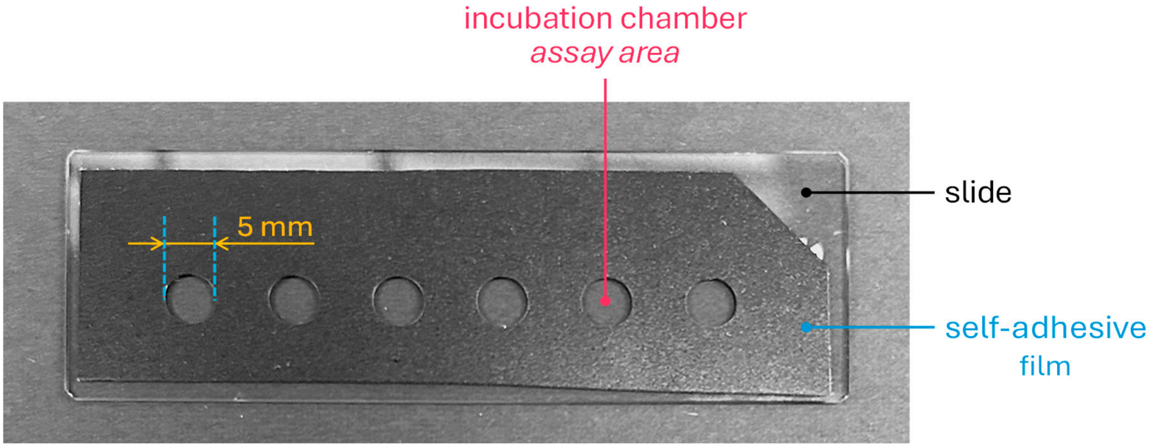

Figure S1. Photograph of the slide's surface before the assay (made of glass or polycarbonate) with six assay areas defined by incubation chambers created by circular apertures in self-adhesive film.

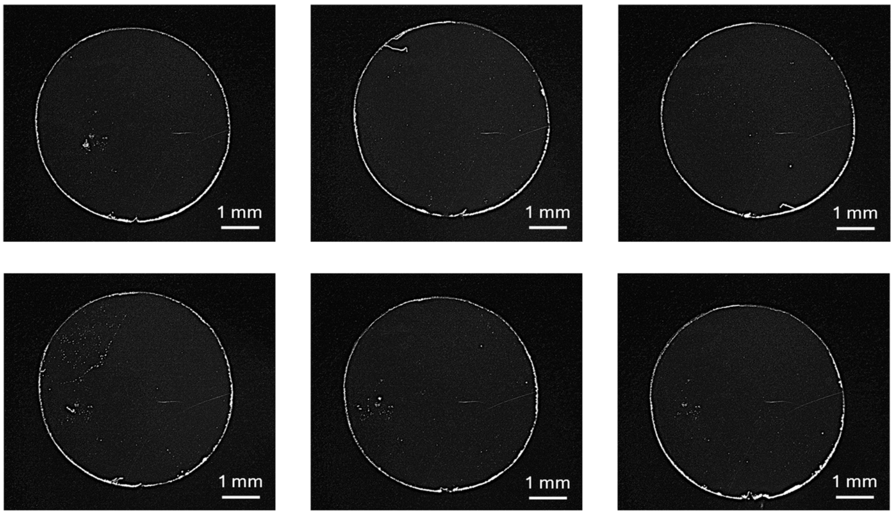

Figure S2. Optical microscopy images of the six replicates of the glass slide surface before the assay.

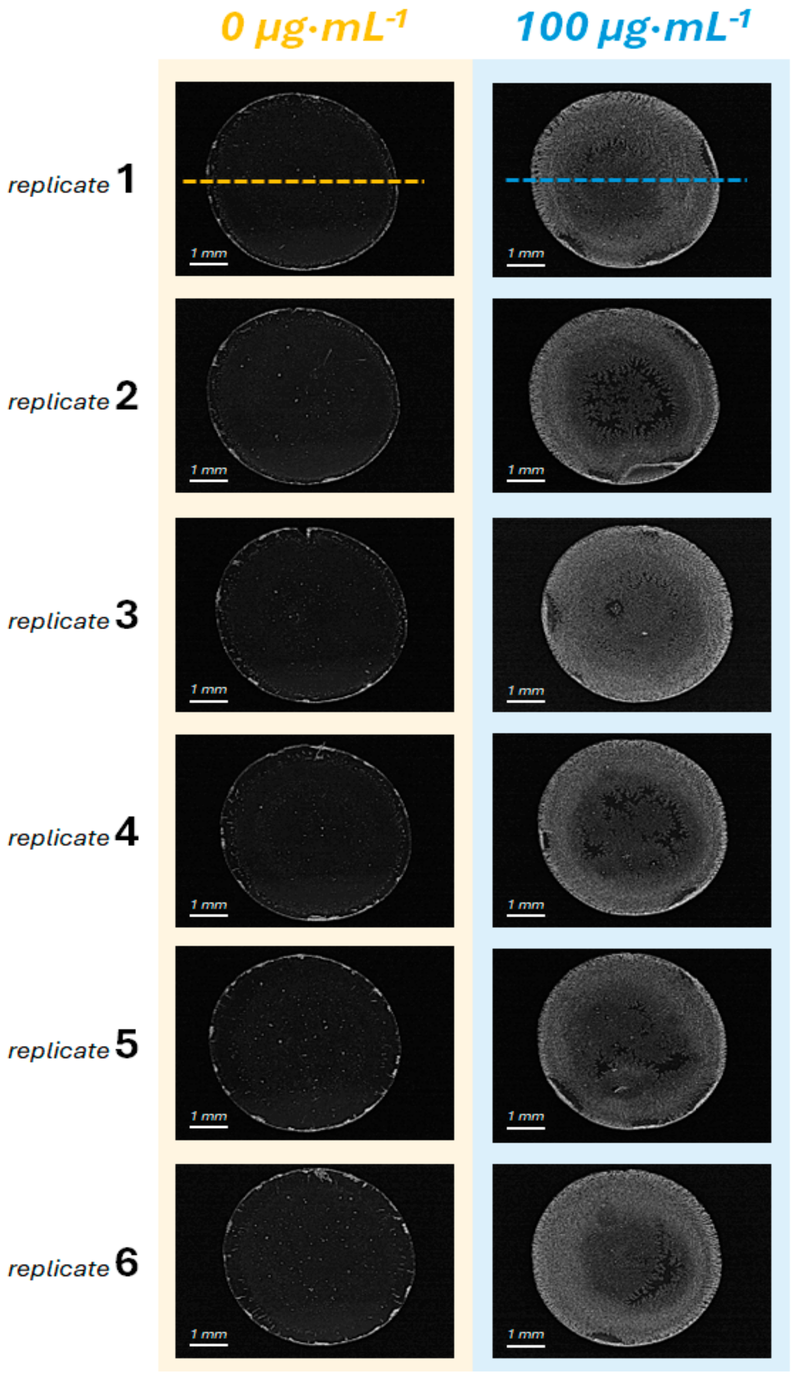

Figure S3. Optical microscopy images of the replicates in the assay shown in Figure1, consisting of glass surfaces functionalized with BSA, after the incubation and drying of anti-BSA antiserum with 0 (left column) and 100 $\mu g \cdot mL^{-1}$ (right column) of specific antibodies in PBS-T. Dashed lines in replicate 1 indicate the cross-section profiles represented in Figure 1C.

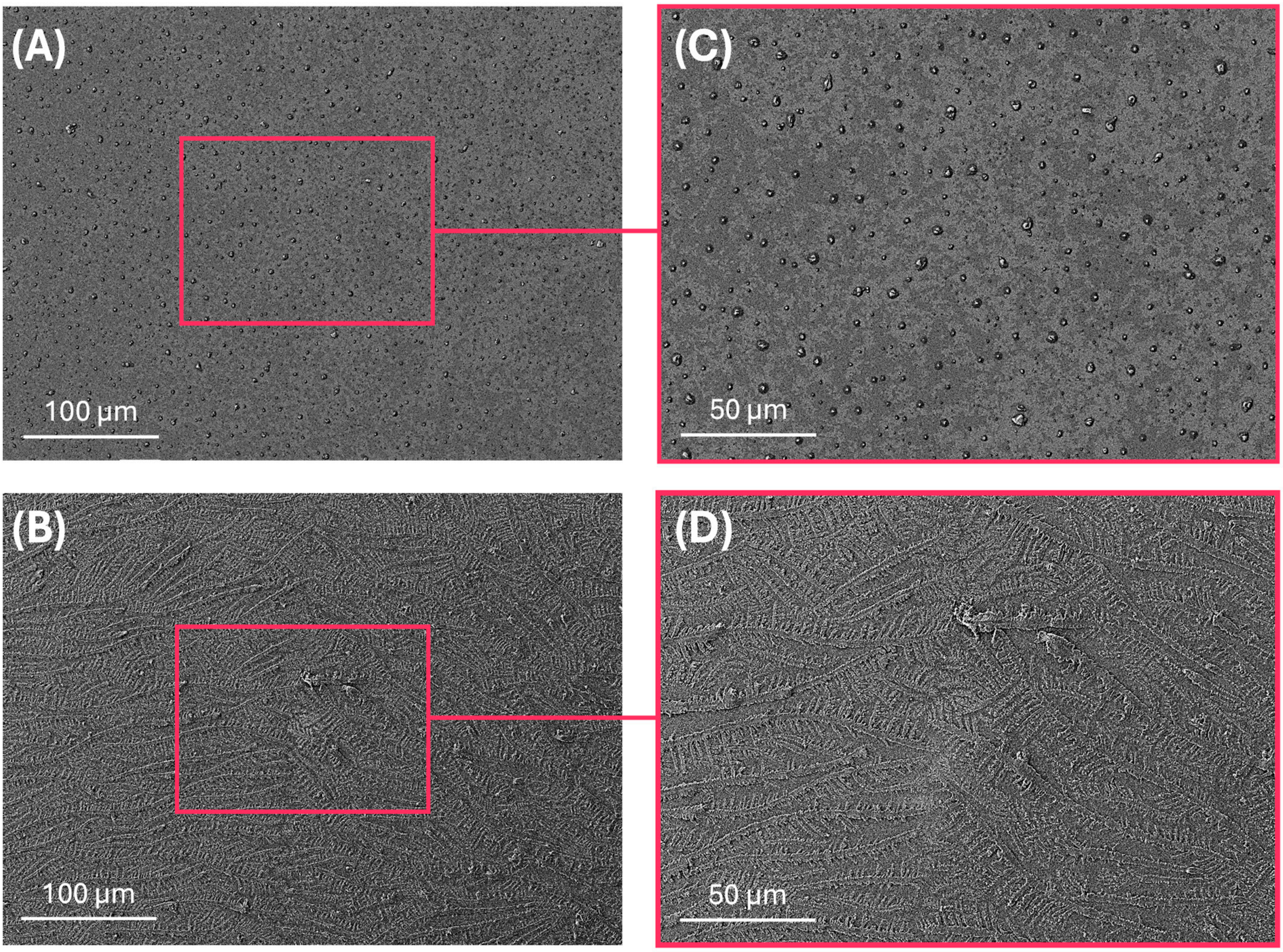

Figure S4. Larger scans of the FESEM images displayed in Figure 2A and 2B. Pictures show larger scans of functionalized BSA-coated slides after the incubation and drying of (A and C) PBS-T and (B and D) of anti-BSA antibodies (100 µg·mL^{-1} in PBS-T). While figures B and D show fractals on the surface, A and C show a random deposition of clusters.

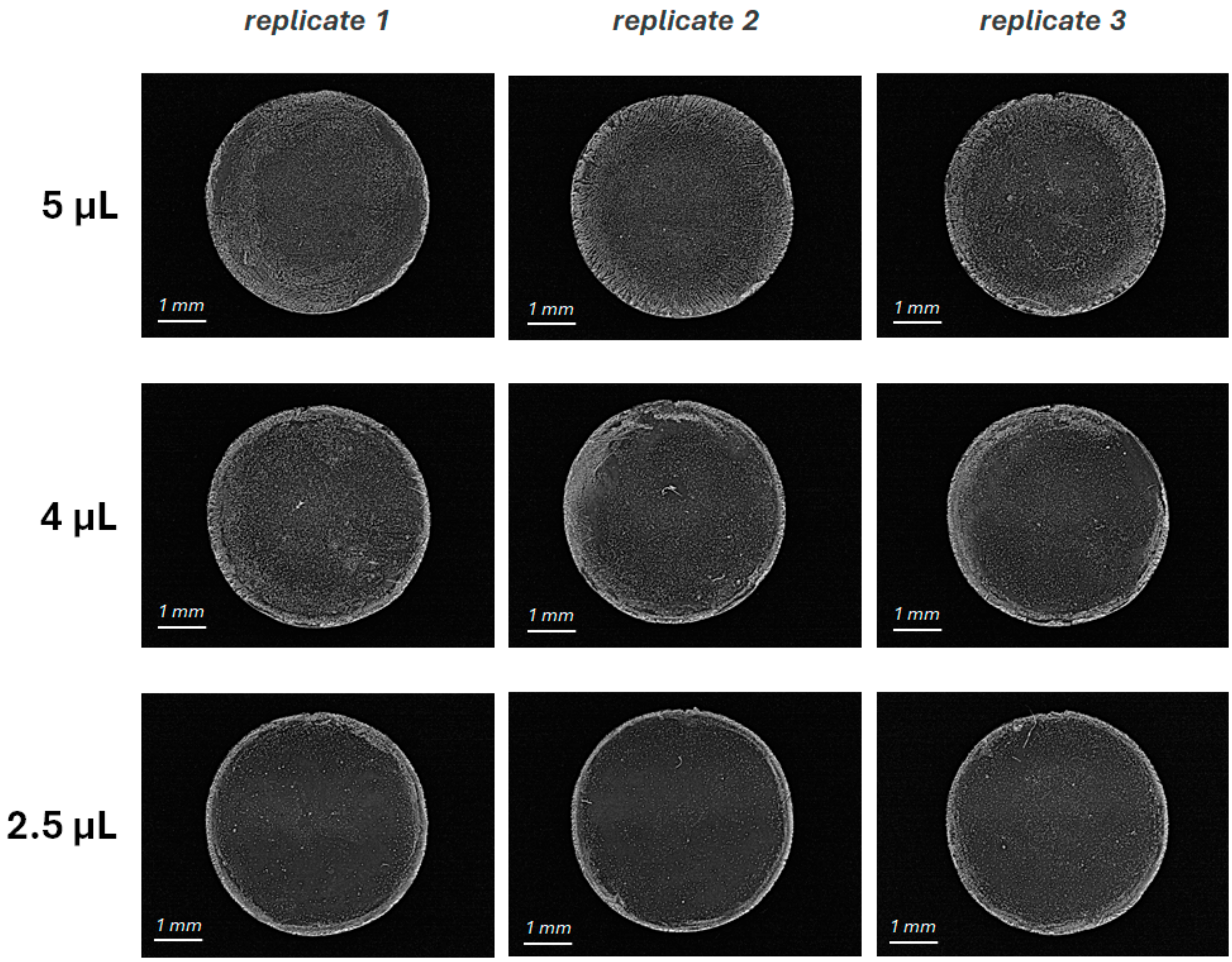

Figure S5. Optical microscopy images of three replicates of assays after the incubation and drying of different volumes (5, 4, and 2.5 µL) of anti-BSA antibodies (100 µg·mL^{-1} in PBS-T) over a glass substrate. The magnitude of the salt deposition in the assays incubated with 2.5 µL of sample is slightly lower.

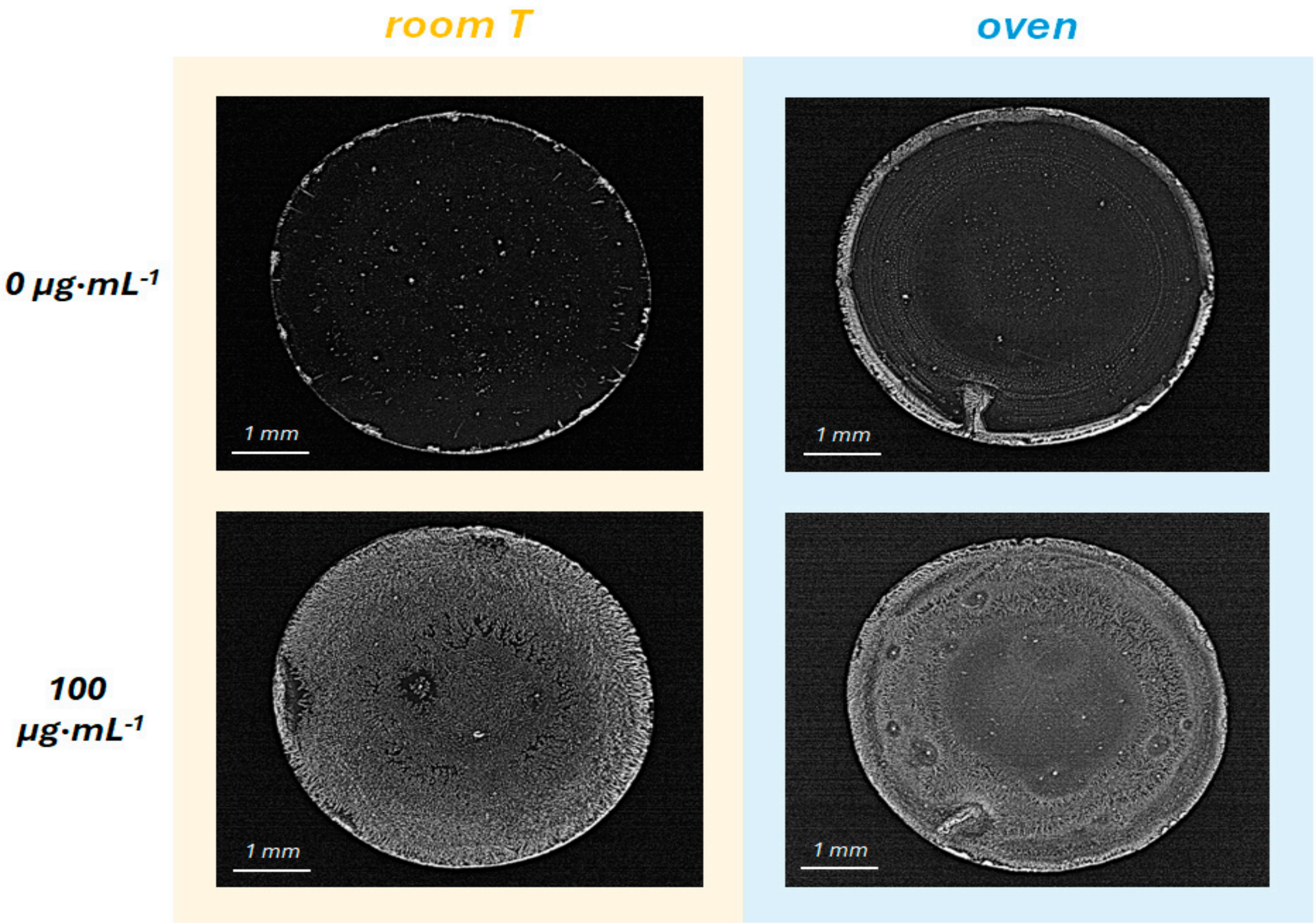

Figure S6. Optical microscopy images of representative assays after the incubation of antiserum with 0 (top) and 100 μg·mL^{-1} (bottom) of IgG in PBS-T, dried at room temperature (left) and in an oven at 37ºC (right).

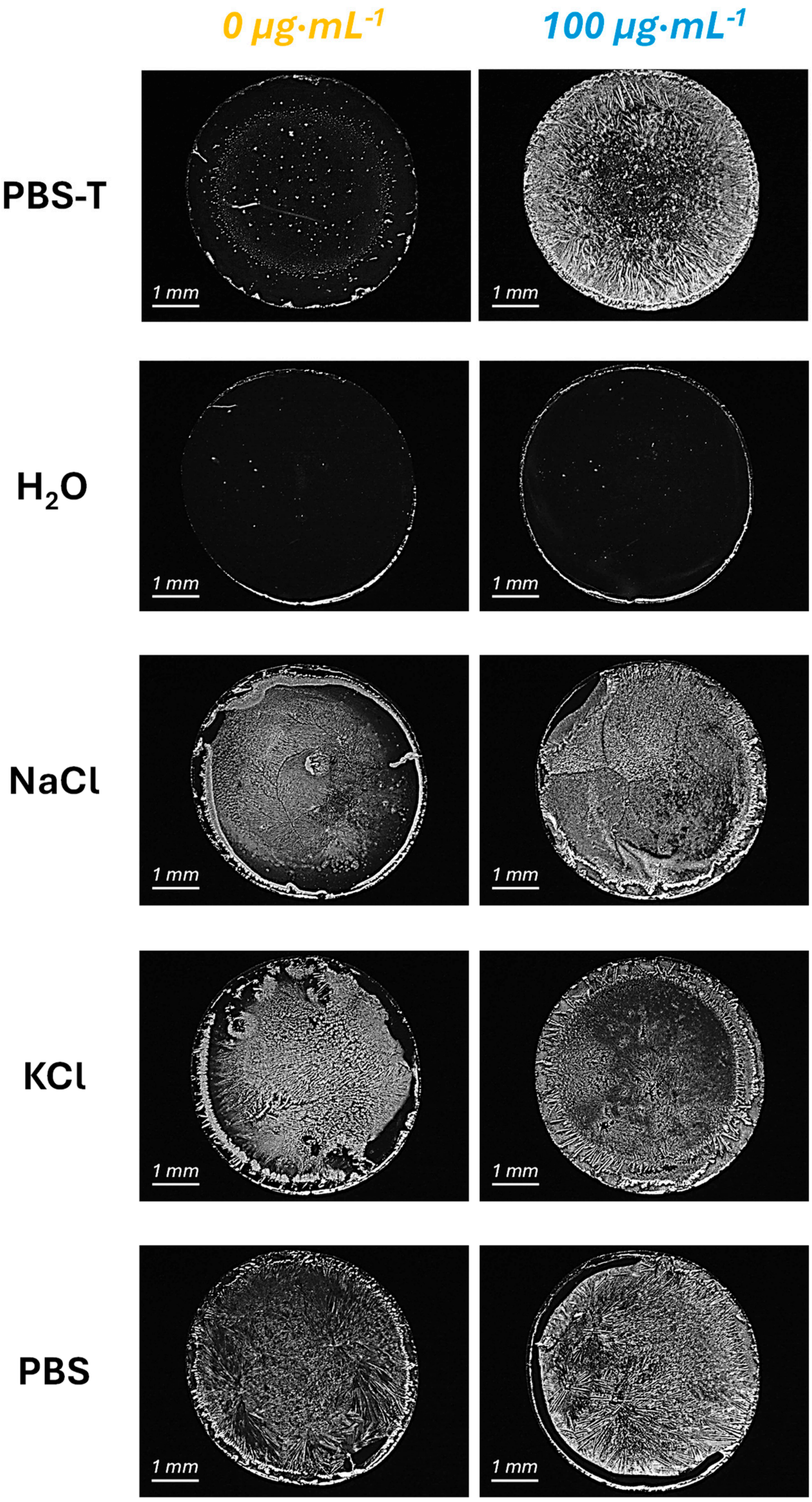

Figure S7. Optical microscopy photographs of assays after the incubation and drying of antiserum with 0 and 100 $\mu g \cdot mL^{-1}$ of IgG solved in different solutions.

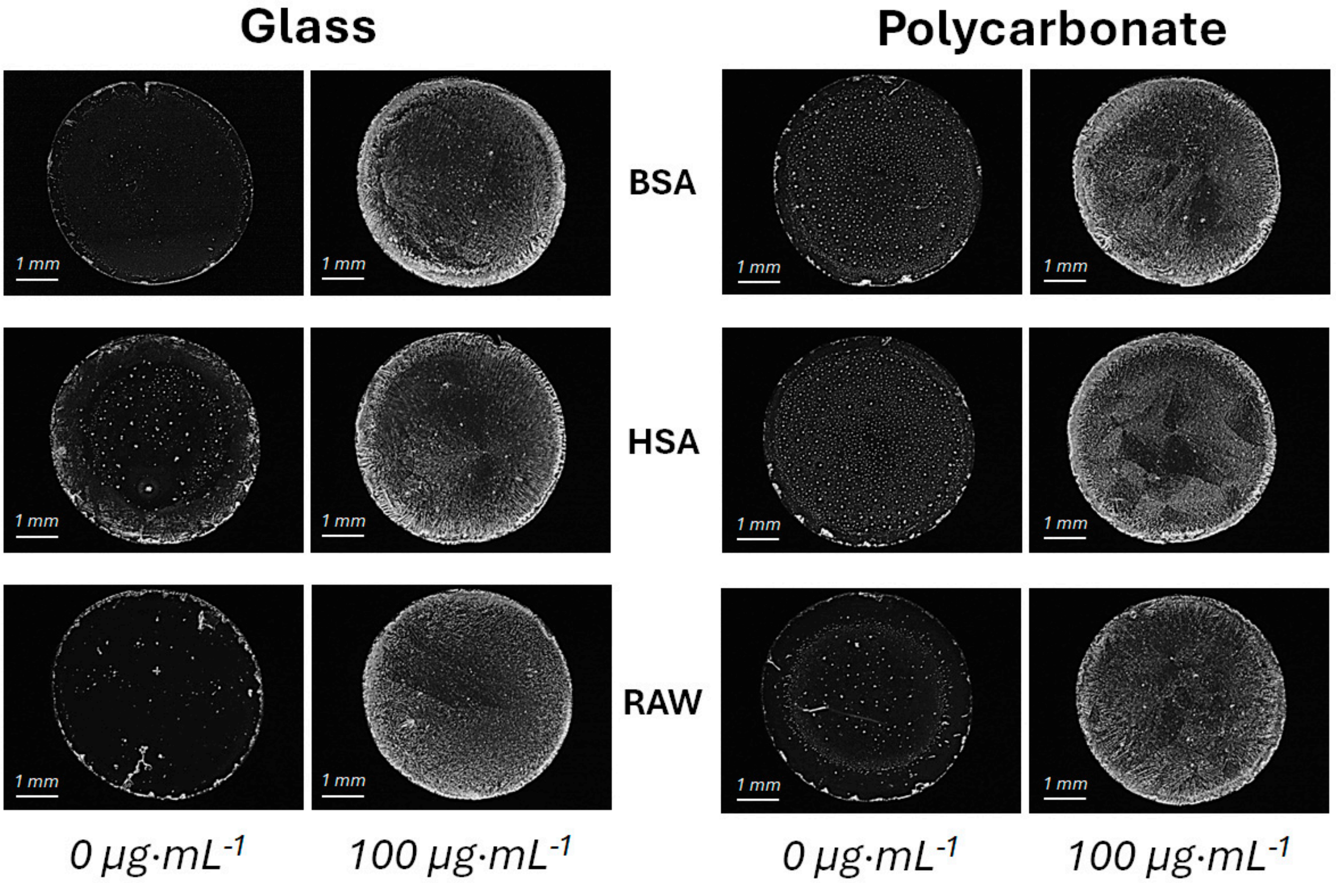

Figure S8. Optical microscopy photographs of representative assays after the incubation and drying of antiserum (0 and 100 µg·mL^{-1} of anti-BSA IgG in PBS-T) on glass and polycarbonate surfaces coated with BSA (top), HSA (middle) and raw materials without any protein coating (bottom).

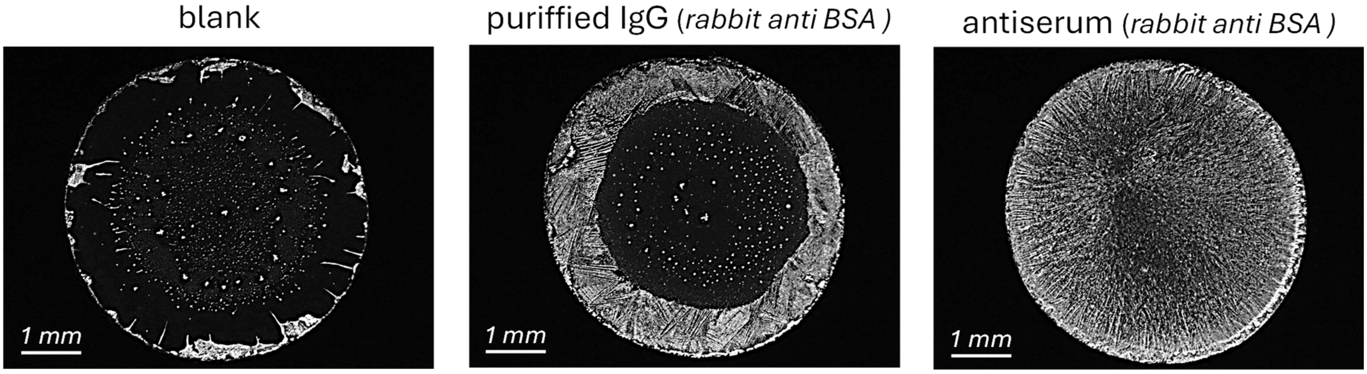

Figure S9. Optical microscopy photographs of representative assays after the incubation of a blank sample (PBS-T), a solution of purified anti-BSA IgG (100 µg·mL^{-1} in PBS-T), and anti-BSA antiserum in PBS-T (100 µg·mL^{-1} of anti-BSA IgG and 950 µg·mL^{-1} of total protein content).

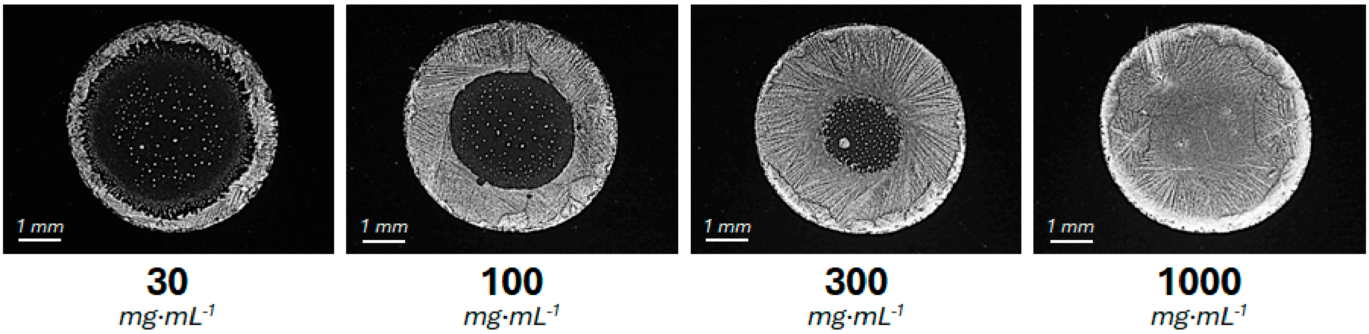

Figure S10. Optical images of assay results for human serum samples at different total protein concentrations, to highlight the radial-growing component that this deposition presents, from the edge to the center with increasing protein concentrations.

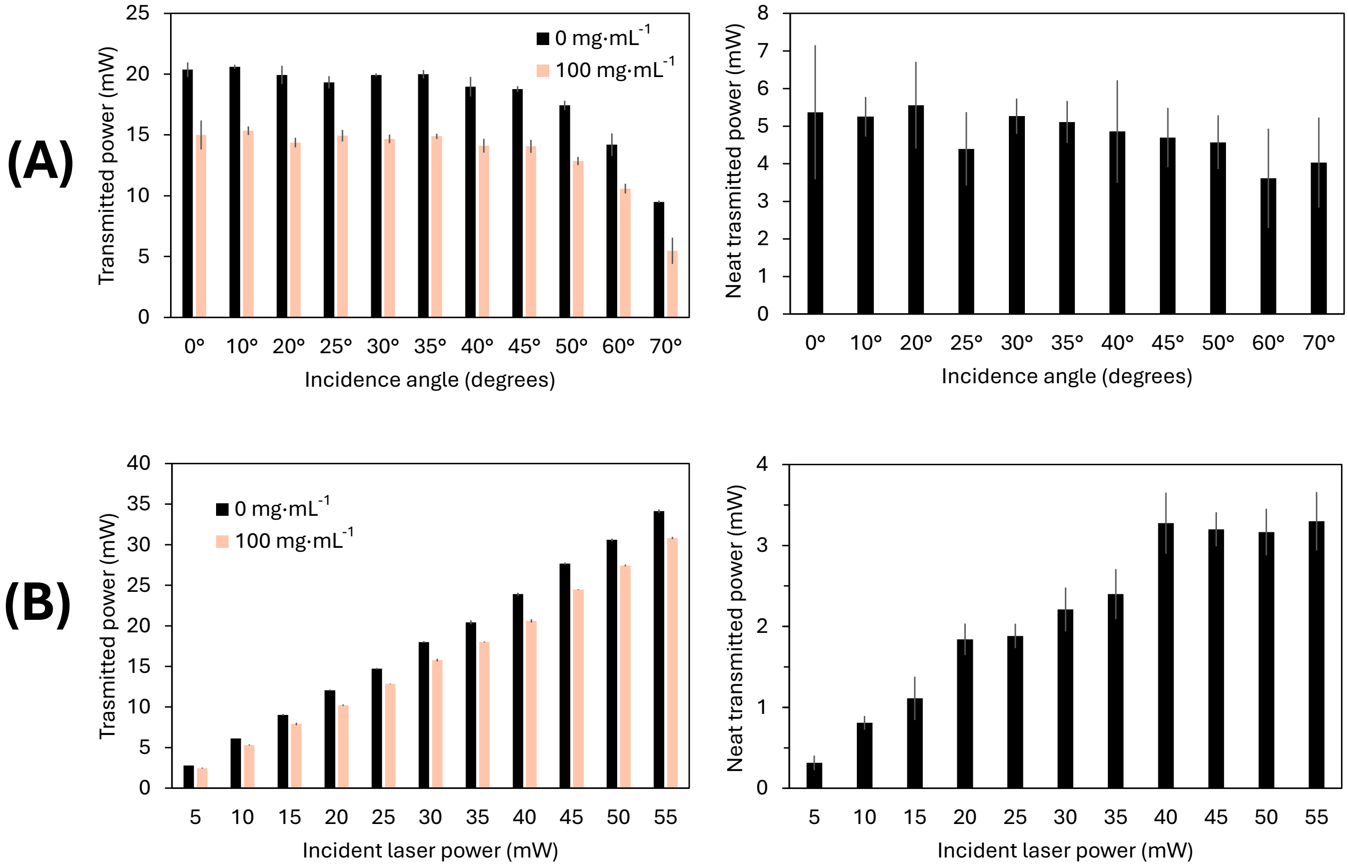

Figure S11. Optimization results of the scattering-based optical setup: (A) Incident angle and (B) power of the incident laser beam. Left graphs display the response after the assay of both 0 and 100 mg·mL^{-1} of anti-BSA in PBS-T. Right graphs represent the neat signal, calculated by subtracting the signal of 100 mg·mL^{-1} from the signal of 0 mg·mL^{-1} at each condition.
